# Metabolites Produced by an Endophytic *Phomopsis* sp. and Their Anti-TMV Activity

**DOI:** 10.3390/molecules22122073

**Published:** 2017-11-27

**Authors:** Qing-Wei Tan, Pei-Hua Fang, Jian-Cheng Ni, Fangluan Gao, Qi-Jian Chen

**Affiliations:** 1 Key Laboratory of Bio-Pesticide and Chemistry-Biology, Ministry of Education, Fujian Agriculture and Forestry University, Fuzhou 350002, China; fjqzfph320@163.com (P.-H.F.); njc2130215001@163.com (J.-C.N.); raindy@fafu.edu.cn (F.G.); 2 Key Laboratory of Plant Virology of Fujian Province, Institute of Plant Virology, Fujian Agriculture and Forestry University, Fuzhou 350002, China

**Keywords:** *Phomopsis*, dothiorelone, cytosporone, *Tobacco mosaic virus*

## Abstract

The fermentation and isolation of metabolites produced by an endophytic fungus, which was identified as *Phomopsis* sp. FJBR-11, based on phylogenetic analysis, led to the identification of six compounds, including dothiorelones A–C, and H, and cytosporones C and U. Among these compounds, cytosporone U exhibited potent inhibitory activity against *Tobacco mosaic virus* (TMV). Moreover, the crude and a purified exopolysaccharide were proved to possess strong inhibitory effects against the virus infection.

## 1. Introduction

*Tobacco mosaic virus* (TMV) is a prevalent plant pathogen which brings about dramatic losses to modern agriculture. Over 885 plant species in 65 families could be infected by TMV, and up to U.S. $100 million is lost each year globally due to plant diseases caused by TMV. However, no effective chemical treatments appear capable, so far, of inhibiting virus replication once plants are infected [1,2,3,4]. As the type member of the genus *Tobamovirus*, TMV is a most commonly used target for discovery of bioactive antiviral natural products.

Plant-associated microorganisms are known to produce a variety of metabolites with novel structures and interesting biological activities [5,6,7]. We have recently reported the anti-TMV activity of malformin A1, which is a cyclic peptide produced by an endophytic fungus obtained from *Brucea javanica* (L.) Merr (Simaroubaceae) [8]. In our ongoing effort to identify antiviral natural products produced by endophytic fungus, a *Phomopsis* strain was isolated from this plant and selected for further study. *Phomopsis* spp. (teleomorph *Diaporthe*) figure among the most common endophytes of tropical plants and some species are the causal agents of important plant diseases. As dominant endophytes, *Phomopsis* spp. have been subjected to intense chemical investigation and various new secondary metabolites have been described, including polyketides, terpenes, and compounds of mixed origin, such as cytochalasins [9,10,11,12,13,14]. We report in this paper the identification of bioactive metabolites produced by an endophytic *Phomopsis* sp. FJBR-11 isolated from *B. javanica*, as well as their anti-TMV activity.

## 2. Results and Discussion

**Figure 1:**
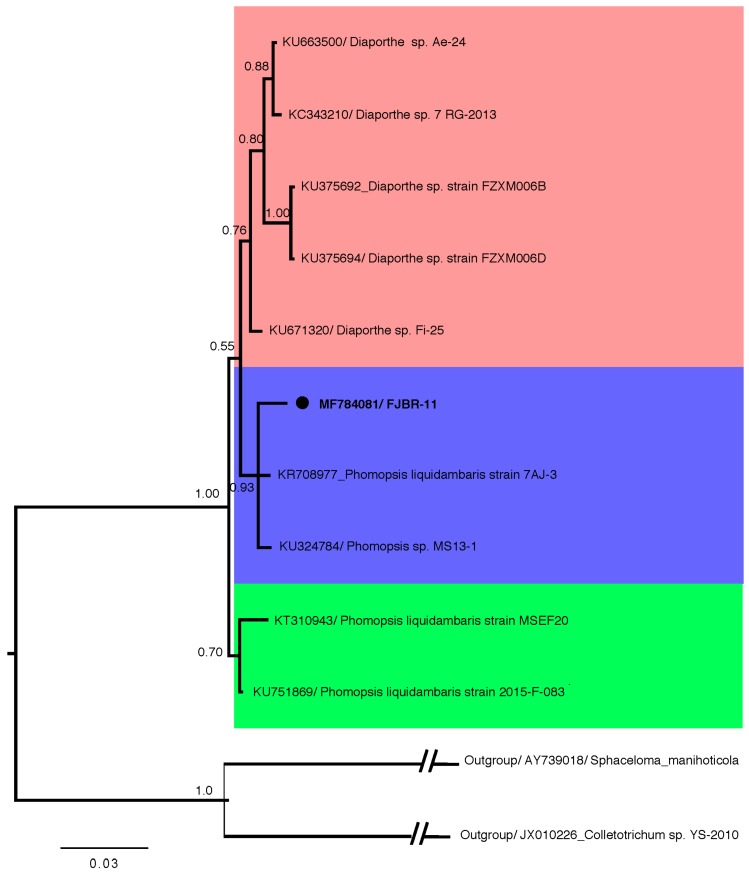
Phylogenetic relationship of isolate FJBR-11 (accession no. MF784081) to representative sequences of known *Phomopsis* sp. and *Diaporthe* sp. Reference sequences were retrieved from GenBank and a *Sphaceloma* sp. and a *Colletotrichum* sp. served as an outgroup. The isolate FJBR-11 from the current study is marked with a black dot. The distance unit is substitutions/site.

Fungal strain FJBR-11 was isolated from the healthy stem tissue of *B. javanica*. The result from Bayesian phylogenetic analysis (Figure 1), based on ITS sequences revealed that FJBR-11 (539 base pairs, GenBank accession no. MF784081) was clustered with high posterior probability (PP = 93) among members of the *Phomopsis* sp., including *Phomopsis liquidambaris* 7AJ-3 (GenBank accession no. KR708977) and *Phomopsis* sp. MS 13-1 (GenBank accession no. KU324784). This suggests that FJBR-11 should be classified as the genus *Phomopsis*.

**Figure 2:**
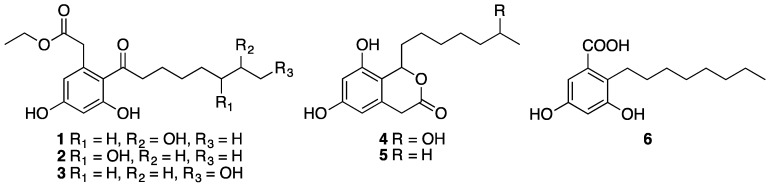
Structure of compounds **1**–**6**.

Fermentation of the fungus *Phomopsis* sp. FJBR-11 and chromatographic fractionation of the EtOAc extract resulted in the isolation of compounds **1**–**6**. Their structures (Figure 2) were identified by comparison of the ^1^H- and ^13^C-NMR data with those previously reported in the literature as dothiorelone A (**1**) [15], dothiorelone B (**2**) [16], dothiorelone C (**3**) [15], dothiorelone H (**4**) [17], cytosporone C (**5**) [18,19], and cytosporone U (**6**) [20]. Compounds **1**–**6** were tested for their antiviral activity against *Tobacco mosaic virus* (TMV) at a concentration of 500 μg/mL using both the local lesion and leaf-disc methods. All the isolated compounds, except cytosporone U (**6**), showed only weak or no inhibitory effect against TMV (Table 1). Cytosporone U showed a dose-dependent inhibition against TMV, and its IC_50_ value was further determined as 144.6 μg/mL using leaf-disc method.

**Figure 3:**
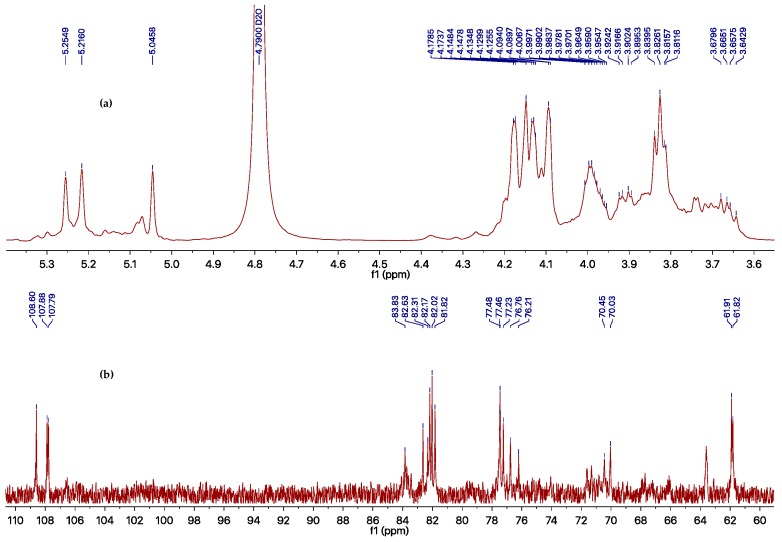
^1^H- (**a**) and ^13^C- (**b**) NMR spectra of impure galactan in D_2_O.

**Figure 4:**
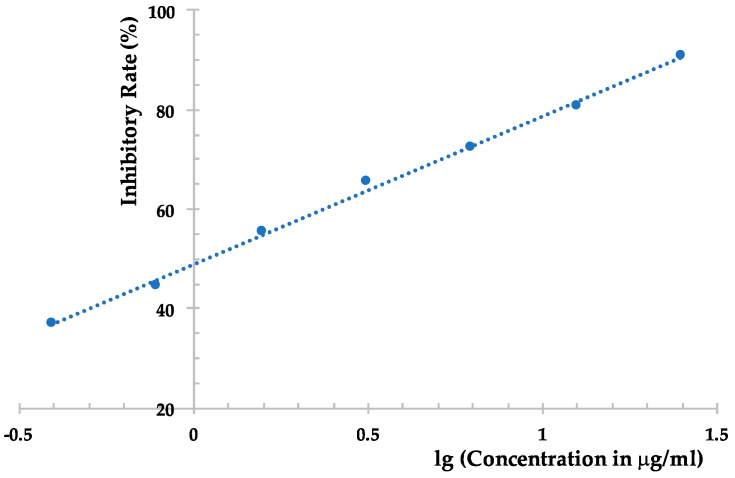
Dose-dependent inhibitory effect of the purified exocellular polysaccharide from the fermentation product of *Phomopsis* sp. FJBR-11 against TMV.

However, the EtOAc extract was more effective than the compounds obtained, which possesses an inhibition rate of >95% at 500 μg/mL against TMV, as determined using the local lesion method. The above results indicated that compounds **1**–**6** could not represent the major antiviral component produced by *Phomopsis* sp. FJBR-11. Our further effort led to the purification of an active exocellular polysaccharide (EP) fraction. The average molecular weight (Mw) was determined as 3.71 × 10^5^ g/mol using gel permeation chromatography (GPC). The chemical structure of the more abundant polysaccharide of this fraction was characterized by means of ^1^H- and ^13^C-NMR, HSQC, TOCSY, as well as NOESY experiments. Its ^13^C-NMR spectra displayed three anomeric carbon signals with similar intensity at *δ*_H_ 5.25, 5.22, and 5.05, which indicated that all these monosaccharides are β-d-Gal*f*, and the polymer consists of a trisaccharide repeating unit of the polysaccharide. Assignment of the ^1^H- and ^13^C-NMR signals (Table 2) for different units was made by HSQC and TOCSY experiments. The observed deshielding of the C-6 of unit **A** allows its assignment as a 6-*O*-substituted β-Gal*f* unit, while the C-5 chemical shift of units **B** and **C** are 6-*O*-substituted β-Gal*f* units. The NOESY cross-peak observed between the anomeric proton of unit **C** with H-6 of unit **A**, between anomeric proton of unit **A** with H-6 of unit **B**, and between anomeric proton of unit **B** with H-6 of unit **C** indicating that unit **C** is (1→6)-linked to unit **A**, which is connected via a (1→5) linkage to unit **B**, which is in turn (1→5)-bonded to unit **C**. The full assigned ^1^H- and ^13^C-NMR data and the above analysis were identical to those described for a galactan isolated from the cell-wall of *Neosartorya* fungus [21]. These data indicated the more abundant polysaccharide of our purified EP fraction produced by *Phomopsis* sp. FJBR-11, a galactan structure with the repeating unit: [→6)-β-d-Gal*f*-(1→5)-β-d-Gal*f*-(1→5)-β-d-Gal*f*-(1→]_n_. Moreover, the ^1^H- and ^13^C-NMR spectra (Figure 3, and Supplementary Materials) were identical with those of an impure phytotoxic exopolysaccharide isolated from the culture filtrate of *Phomopsis foeniculi*, as reported in the literature [22], whose chemical structure was also characterized with the same repeating unit described above. The purified EP fraction showed a dose-dependent inhibition (Figure 4) against TMV with an IC_50_ value of 1.08 μg/mL, as determined by the local lesion method, but no inhibitory effect was observed when tested using the leaf-disc method at a concentration of 500 μg/mL.

Polysaccharides have many kinds of biological activities, such as anti-oxidation, hypoglycemic, enhancing immunity, anti-aging, anti-rheumatism, anti-cancer, and anti-viral properties. etc., which indicating a wide application prospect for their use in the field of medicine and pesticides [23,24]. The production of polysaccharides inducing phytotoxic effects was reported for several fungal pathogens, among which mycolaminarans from the cytoplasma of *Phytophthora palmivora*, *P. cinnamomi*, and *P. megasperma* var. sojae [25], and EP from a culture filtrate of *P. cinnamomi*, *P. cryptogea*, and *P. nicotianae* [26] could induce severe wilting on several hosts. More recently, the crude polysaccharide fraction produced by *P. foeniculi* and its components, galactan and manan, were reported showing phytotoxic effects, i.e., chlorosis, necrosis, and/or wilting, on fennel and on two non-host plants, tobacco and tomato. The macromolecules commonly appear to act by interfering with water movement in plant tissues due to mechanical plugging of the vessels which leads to wilt symptoms, and the phenomenon appears to be related to the size of the molecules and their viscosity rather than to their structure [21]. The exopolysaccharide produced by *Phomopsis* sp. FJBR-11 was found possessing a high inhibitory activity against TMV infection in our present study. A further investigation on the relationship between the antiviral effect and the size and viscosity of the polysaccharide will help us understand its role and behavior.

## 3. Materials and Methods

### 3.1. General Experimental Procedures

^1^H- and ^13^C-NMR spectra were obtained with a Bruker AVANCE III 500 spectrometer (Bruker BioSpin, Rheinstetten, Germany) and a JEOL JNM-ECA400 spectrometer (JEOL Ltd., Tokyo, Japan) using tetramethylsilane as an internal standard. Sephadex LH-20 (25–100 μm, Pharmacia Fine Chemical Co., Ltd., Uppsala, Sweden), Lichroprep RP-18 gel (40–63 μm, Merck, Darmstade, Germany), silica gel (200–300 mesh) and silica gel H (Qingdao Oceanic Chemical Co., Qingdao, China) were used for column chromatography. Thin-layer chromatography (TLC) was performed on glass-backed plates coated with 0.25 mm layers of silica gel H (Qingdao Oceanic Chemical Co., Qingdao, China). Fractions were monitored by TLC and spots were visualized by heating silica gel plates sprayed with 5% H_2_SO_4_ in EtOH. All solvents and chemicals used were of analytical reagent grade (Sinopharm Chemical Reagent Co., Ltd., Beijing, China), and water was doubly distilled before use.

### 3.2. Fungal Strain

The endophytic fungus FJBR-11 was isolated from the healthy stems of *B. javanica* collected from the Xiamen Overseas Chinese Subtropical Plant Introduction Garden (Xiamen, China) in 2014. The pure culture is deposited in the Key Laboratory of Bio-Pesticide and Chemistry-Biology, Ministry of Education, Fujian Agriculture and Forestry University, Fuzhou, China. This fungal colony on PDA was white and did not produce any spores. Therefore, it was identified by molecular technique based on the DNA sequence data of the internal transcribed spacer (ITS1 and ITS4) regions of the ribosomal RNA gene using universal fungal primers.

The nucleotide (nt) sequences were aligned using the MUSCLE algorithm [27] implemented in MEGA5 [28]. The degree of mutational saturation in the aligned sequences was evaluated using the Iss statistic in DAMBE 6.0 [29]. Bayesian phylogenetic analysis using MrBayes 3.2.6 [30] under the *K80 + G* model, which was determined by jModelTest 2.17 [31]. Four Markov Monte Carlo (MCMC) chains were run for 2,000,000 generations, sampling every 100 generations, with the final standard deviation of split frequencies <0.01 and the first 25% of sampled trees discarded as burn-in. The Bayesian 50% majority rule consensus tree was visualized in FigTree 1.4.3.

### 3.3. Fermentation, Extraction, and Isolation

The *Phomopsis* fungus FJBR-11 was grown on potato dextrose agar (PDA) at 28 °C for seven days. Three pieces (0.5 × 0.5 cm) of mycelial agar plugs were inoculated into 500 mL Erlenmeyer flasks containing 200 mL of potato dextrose broth (PDB) at 28 °C on a rotary shaker at 120 rpm for seven days to obtain the seed culture. Cultivation was carried out in a 50 L fermentor containing 30 L of liquid PDB at 28 °C for three weeks. The fermentation broth was harvested and filtrated. The filtrate was evaporated in vacuo to give a crude solution of 2 L, which was then extracted with EtOAc (500 mL × 3) to give an EtOAc soluble fraction (19.8 g). The EtOAc fraction was dissolved with 60% MeOH in H_2_O, and separated by column chromatography over Lichroprep RP-18 and eluted with 60% MeOH to 100% MeOH in H_2_O to give 18 fractions (Fractions 1–18). Fraction 5 was subjected to Sephadex LH-20 column chromatography and eluted with a mixture of CHCl_3_ and MeOH (*v*/*v*, 1:1) to give compound **4** (14.0 mg). Fraction 11 (118 mg) was subjected to silica column chromatography using a mixture of petroleum ether and acetone (*v*/*v*, 70:30), and further purified with Sephadex LH-20 column chromatography to give compounds **1** (9.8 mg), **2** (24.3 mg), and **3** (6.4 mg). Fraction 14 (153 mg) was subjected to Sephadex LH-20 column chromatography and eluted with a mixture of CHCl_3_ and MeOH (*v/v*, 1:1) to give two subfractions, which were then subjected to silica column chromatography using a mixture of CHCl_3_ and MeOH (*v*/*v*, 98:2) and CHCl_3_ and MeOH (*v*/*v*, 95:5), respectively, to give compounds **5** (20.0 mg) and **6** (15.0 mg).

Another 15 L fermentation broth was obtained under the same culture conditions as described above. The filtrate was evaporated *in vacuo* to give a crude solution of 1 L, which was then subject to column chromatography over Diaion HP-20 and eluted with 0%, 25%, 50%, 75%, and 100% MeOH in H_2_O (*v*/*v*). The active fraction (25% MeOH elute), was subjected to silica gel column chromatography and eluted with a gradient EtOH in H_2_O (100%, 95%, 90%, 80%, 70%, 60%, and 50%). The 80% EtOH elute (280 mg) and 70% EtOH elute (45 mg) were both crude polysaccharide and showed >95% inhibition against TMV as tested using local lesion method under concentration of 500 μg/mL. The 70% EtOH elute was further purified using column chromatography over a Sephadex LH-20, eluted with H_2_O to give a polysaccharide fraction (18 mg) for NMR testing.

#### 3.3.1. Dothiorelone A (**1**)

Colorless gum; ^1^H-NMR (400 MHz, CD_3_OD) δ 6.24 (1H, d, *J* = 2.3 Hz, H-6), 6.18 (1H, d, *J* = 2.3 Hz, H-4), 4.10 (2H, q, *J* = 7.1 Hz, H-17), 3.68 (1H, m, H-15), 3.57 (2H, s, H-2), 2.88 (2H, t, *J* = 7.5 Hz, H-10), 1.61 (2H, m, H-11), 1.40 (6H, m, H-12, 13, 14), 1.23 (3H, t, *J* = 7.1 Hz, H-18), 1.13 (3H, d, *J* = 6.2 Hz, H-16). ^13^C-NMR (100 MHz, CD_3_OD) δ 209.0 (C-9), 173.6 (C-1), 161.4 (C-5), 159.8 (C-7), 137.1 (C-3), 121.2 (C-8), 111.7 (C-4), 102.7 (C-6), 68.5 (C-15), 61.9 (C-17), 45.2 (C-10), 40.5 (C-2), 40.1 (C-14), 30.6 (C-13), 26.8 (C-12), 25.5 (C-11), 23.5 (C-16), 14.5 (C-18).

#### 3.3.2. Dothiorelone B (**2**)

White solid; ^1^H-NMR (400 MHz, CD_3_OD) δ 6.23 (1H, d, *J* = 2.1 Hz, H-6), 6.16 (1H, d, *J* = 2.0 Hz, H-4), 4.08 (2H, q, *J* = 7.1 Hz, H-17), 3.55 (2H, s, H-2), 3.41 (1H, m, H-14), 2.90 (2H, t, *J* = 7.5 Hz, H-10), 1.60 (2H, m, H-11), 1.31–1.49 (6H, m, H-12, 13, 15), 1.22 (3H, t, *J* = 7.1 Hz, H3-18), 0.90 (3H, t, *J* = 7.4 Hz, H3-16). ^13^C-NMR (100 MHz, CD_3_OD) δ 208.9 (C-9), 173.6 (C-1), 161.4 (C-5), 159.8 (C-7), 137.1 (C-3), 121.2 (C-8), 111.7 (C-4), 102.7 (C-6), 73.8 (C-14), 61.9 (C-17), 45.2 (C-10), 40.6 (C-2), 37.8 (C-13), 31.0 (C-12), 26.6 (C-15), 25.7 (C-11), 14.5 (C-18), 10.4 (C-16).

#### 3.3.3. Dothiorelone C (**3**)

White solid; ^1^H-NMR (400 MHz, CD_3_OD) δ 6.26 (1H, d, *J* = 2.3 Hz, H-6), 6.19 (1H, d, *J* = 2.3 Hz, H-7), 4.11 (2H, q, *J* = 7.1 Hz, H-17), 3.58 (2H, s, H-2), 3.54 (2H, t, *J* = 6.6 Hz, H-16), 2.89 (2H, t, *J* = 7.3 Hz, H-10), 1.61 (2H, m, H-11), 1.52 (2H, m, H-15), 1.35 (6H, m, H-12, H-13, and H-14), 1.24 (3H, t, *J* = 7.1 Hz, H-18). ^13^C-NMR (100 MHz, CD_3_OD) δ 209.0 (C-9), 173.6 (C-1), 161.3 (C-5), 159.9 (C-7), 137.0 (C-3), 121.2 (C-8), 111.8 (C-4), 102.7 (C-6), 63.0 (C-16), 61.9 (C-17), 45.2 (C-10), 40.5 (C-2), 33.6 (C-15), 30.4 (C-12, 14), 26.8 (C-13), 25.5 (C-11), 14.5 (C-18).

#### 3.3.4. Dothiorelone H (**4**)

Pale yellow oil; ^1^H-NMR (500 MHz, CD_3_OD) δ 6.23 (1H, d, *J* = 2.2 Hz, H-6), 6.14 (1H, d, *J* = 2.1 Hz, H-4), 5.61 (1H, dd, *J* = 8.7, 4.9 Hz, H-9), 3.79 (1H, d, *J* = 19.6 Hz, H-2a), 3.70 (1H, m, H-15), 3.48 (1H, d, *J* = 19.6 Hz, H-2b), 1.91–1.75 (2H, m, H-10), 1.63–1.28 (8H, m, H-11, H-12, H-13, and H-14), 1.14 (3H, d, *J* = 6.2 Hz, H_3_-16). ^13^C-NMR (125 MHz, CD_3_OD) δ 174.1 (C-1), 159.5 (C-5), 155.2 (C-7), 132.8 (C-3), 113.9 (C-8), 106.1(C-4), 102.0 (C-6), 80.1 (C-9), 68.5 (C-15), 40.1 (C-14), 36.7 (C-10), 35.5 (C-2), 30.3 (C-12), 26.7 (C-13), 26.6 (C-11), 23.5 (C-16).

#### 3.3.5. Cytosporone C (**5**)

White powder; ^1^H-NMR (500 MHz, CD_3_OD) δ 6.23 (1H, d, *J* = 2.3 Hz, H-6), 6.14 (1H, d, *J* = 2.2 Hz, H-4), 5.61 (1H, dd, *J* = 8.6, 5.2 Hz, H-9), 3.79 (1H, d, *J* = 19.6 Hz, H-2a), 3.48 (1H, d, *J* = 19.6 Hz, H-2b), 1.90–1.74 (2H, m, H-10), 1.53 (1H, m, H-11a), 1.43 (1H, m, H-11b), 1.40–1.24 (8H, m, H-12, H-13, H-14, and H-15), 0.90 (3H, t, *J* = 6.9 Hz, H_3_-16). ^13^C-NMR (125 MHz, CD_3_OD) δ 174.1 (C-1), 159.5 (C-5), 155.2 (C-7), 132.8 (C-3), 113.9 (C-8), 106.0 (C-4), 102.0 (C-6), 80.1 (C-9), 36.7 (C-10), 35.5 (C-2), 32.9 (C-14), 30.3 (C-13), 30.2 (C-12), 26.6 (C-11), 23.7 (C-15), 14.4 (C-16).

#### 3.3.6. Cytosporone U (**6**)

White amorphous powder; ^1^H-NMR (500 MHz, CD_3_OD) δ 6.20 (1H, d, *J* = 2.4 Hz, H-6), 6.19 (1H, d, *J* = 2.4 Hz, H-4), 3.49 (2H, s, H-2), 2.50 (2H, t, *J* = 8.0 Hz, H-9), 1.43 (2H, m, H-13), 1.38–1.22 (10H, overlap, H-10, H-11, H-12, H-14, and H-15), 0.88 (3H, t, *J* = 7.5 Hz, H-16). ^13^C-NMR (125 MHz, CD_3_OD) δ 176.1 (C-1), 157.4 (C-5), 156.5 (C-7), 135.7 (C-3), 121.1 (C-8), 109.7 (C-4), 102.5 (C-6), 39.7 (C-2), 33.1 (C-14), 31.0 (C-13), 30.9 (C-10), 30.6 (C-12), 30.5 (C-11), 26.9 (C-9), 23.7 (C-15), 14.4 (C-16).

### 3.4. Determination of the Molecular Weight of the Polysaccharide

The molecular weight of the polysaccharide was determined by gel permeation chromatography (GPC) with a Waters 515 GPC system (Milford, MA, USA) equipped with light-scattering (LS) detector and differential refractive index (DRI) detector, and an OHpak SB-802.5HQ column. A 20 µL sample was injected, and the mobile phase consisting of 0.2 M NaNO_3_ was used at a flow rate of 0.5 mL/min. The column temperature was set as 25 °C and the operation time was 40 min. PEG was used as the standard sample and established the standard curve.

### 3.5. Anti-TMV Assay

#### 3.5.1. Virus and Host Plant

Purified TMV (strain U1) was obtained from the Institute of Plant Virology, Fujian Agriculture and Forestry University, Fuzhou, China, and the concentration was determined as 15 mg/mL using the ultraviolet spectrophotometer method $\left(\mathrm{virus}\;\mathrm{concentration}=(A_{260}\times \mathrm{dilution}\;\mathrm{ration})/\;E_{1\;\mathrm{cm}}^{0.1\%,\;260\;\mathrm{nm}}\right)$. The purified virus was kept at −20 °C and was diluted to 30 μg/mL with 0.01 M PBS before use. *Nicotiana glutinosa* and *Nicotiana tabacum* cv. K326, which were cultivated and grown to the 5–6 leaf stage in an insect-free greenhouse, were used for anti-TMV assay as the local lesion and systemic TMV infection hosts, respectively. Purified compounds were dissolved in DMSO and diluted with 0.01 M PBS to a certain concentration for testing. The final concentration of DMSO in the test solution (≤2%) was proved to show no adverse effect on the plants.

#### 3.5.2. Local-Lesion Assay

A mixture of test solution with TMV (final concentration of 10 μg/mL) was made 30 min before inoculation. The mixture was inoculated on the left side of the leaves of *N. glutinosa*, while the right side of the same leaves were inoculated with a solution of the negative control mixed with an equal concentration of TMV. All assays were conducted in triplicate. The numbers of the local lesion were recorded 3–4 d after inoculation, and inhibition rates were calculated according to the following formula: Inhibition rate (%) = ((*C* − *T*)/*C*]) × 100%, where *C* is the average number of local lesions of the control, while *T* is the average number of local lesions of the treatment.

#### 3.5.3. Leaf-Disc Method

Growing leaves of *N. tabacum* cv. K326 were mechanically inoculated with TMV (30 μg/mL in 0.01 M PBS). After 6 h, leaf discs (1 cm diameter) were punched and floated on solutions for testing. Discs of healthy and inoculated leaves floated on a solution of 0.01 M PBS with 2% DMSO were used as mock and negative controls, respectively. Three replicates were carried out for each sample. After incubating for 48 h at 25 °C in a culture chamber, the leaf discs were ground in 0.01 M carbonate coating buffer (pH 9.6) and OD_405_ values were measured using the TAS-ELISA method. TAS-ELISA was performed as described in the literature [32,33]. Virus concentration was calculated from a standard curve constructed using OD_405_ values of purified TMV at concentrations of 1.0, 0.5, 0.25, 0.125, and 0.0625 μg/mL. The inhibition of test solutions on TMV was calculated as follows: Inhibition rate = (1 − (virus concentration of treatment)/(virus concentration of negative control)) × 100%.

## 4. Conclusions

Six compounds, including dothiorelones A–C and H, and cytosporones C and U, were identified from the metabolites of an endophytic *Phomopsis* sp. FJBR-11 isolated from the stem of *B. javanica*. All the compounds, except cytosporone U, showed only weak or no inhibitory activity against TMV under test using the local lesion and leaf-disc methods. However, the crude and a purified polysaccharide produced by *Phomopsis* sp. FJBR-11 showed strong inhibitory effects against the virus infection as determined by using the local lesion assay.

## Figures and Tables

**Table 1:** Antiviral activity of Compounds **1**–**6** (500 μg/mL) against TMV.

Compound	Inhibition Rate (%)	
	Local Lesion Assay	Leaf-Disc Method
**1**	<10	<10
**2**	<10	10.9 ± 1.1
**3**	<10	12.7 ± 1.0
**4**	11.3 ± 1.2	29.6 ± 2.1
**5**	22.9 ± 2.1	31.9 ± 2.0
**6**	39.2 ± 2.2	80.0 ± 3.0
Ningnanmycin	90.3 ± 3.4	91.4 ± 3.8

**Table 2:** ^1^H- and ^13^C-NMR chemical shifts for the impure galactan from *Phomopsis* sp. FJBR-11.

Position	Unit A		Unit B		Unit C	
	*δ*_C_ (ppm)	*δ*_H_ (ppm)	*δ*_C_ (ppm)	*δ*_H_ (ppm)	*δ*_C_ (ppm)	*δ*_H_ (ppm)
1	107.8	5.25	107.9	5.22	108.6	5.05
2	82.2	4.17	82.0	4.18	81.8	4.15
3	77.5	4.09	77.5	4.13	77.2	4.13
4	83.8	4.09	82.3	4.15	82.6	4.13
5	70.4	4.02–3.95	76.2	4.02–3.95	76.8	4.02–3.95
6	70.0	3.93–3.89, 3.69–3.64	61.9	3.85–3.80	61.8	3.85–3.80

## Supplementary Materials

Supplementary materials are available online. ^1^H- and ^13^C-NMR spectra of compounds **1**–**6**; ^1^H- and ^13^C-NMR spectra, as well as HSQC, TOCSY, and NOESY spectra of the purified polysaccharide.
