# Supplementary material for: Metabolites Produced by an Endophytic Phomopsis sp. and Their Anti-TMV Activity
Source: Molecules. 2017 Nov 27;22(12):2073. doi: 10.3390/molecules22122073 (PMC6149851; doi:10.3390/molecules22122073)

Sulpplementary Materials

Title: Metabolites Produced by an Endophytic *Phomopsis* sp. and Their Anti-TMV Activity

Author(s): Qing-Wei Tan *, Pei-Hua Fang, Jian-Cheng Ni, Fangluan Gao and Qi-Jian Chen *

S1. ^{1}H-NMR spectrum (400 MHz) of Dothiorelone A (1) in CD_3OD.

S2. ^{13}C-NMR spectrum (100 MHz) of Dothiorelone A (1) in CD_3OD.

S3. ^{1}H-NMR spectrum (400 MHz) of Dothiorelone B (2) in CD_3OD.

S4. ^{13}C-NMR spectrum (100 MHz) of Dothiorelone B (2) in CD_3OD.

S5. ^{1}H-NMR spectrum (400 MHz) of Dothiorelone C (3) in CD_3OD.

S6. ^{13}C-NMR spectrum (100 MHz) of Dothiorelone C (3) in CD_3OD.

S7. ^{1}H-NMR spectrum (500 MHz) of Dothiorelone H (4) in CD_3OD.

S8. ^{13}C-NMR spectrum (125 MHz) of Dothiorelone H (4) in CD_3OD.

S9. ^{1}H-NMR spectrum (500 MHz) of Cytosporone C (5) in CD_3OD.

S10. ^{13}C-NMR spectrum (125 MHz) of Cytosporone C (5) in CD_3OD.

S11. ^{1}H-NMR spectrum (500 MHz) of Cytosporone U (6) in CD_3OD.

S12. ^{13}C-NMR spectrum (125 MHz) of Cytosporone U (6) in CD_3OD.

S13. ^{1}H-NMR spectrum (500 MHz) of the purified polysaccharide in D_2O.

S14. ^{13}C-NMR spectrum (125 MHz) of the purified polysaccharide in D_2O.

S15. HSQC spectrum of the purified polysaccharide.

S16. TOSCY spectrum of the purified polysaccharide.

S17. NOESY spectrum of the purified polysaccharide.

S1. ^{1}H-NMR spectrum (400 MHz) of Dothiorelone A (1) in CD_3OD.

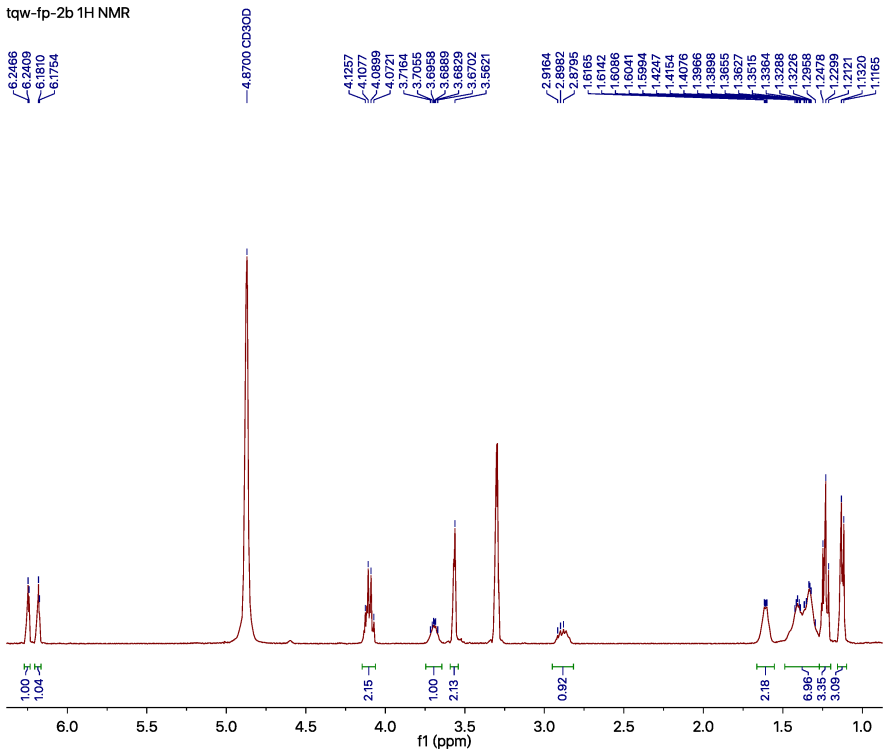

	Parameter	Value
1	Title	FP_2b
2	Origin	JEOL
3	Spectrometer	ECA
4	Author	delta
5	Solvent	METHANOL_D4
6	Temperature	22.1
7	Pulse Sequence	proton.jxp
8	Experiment	1D
9	Probe	2756
10	Number of Scans	8
11	Receiver Gain	56.0
12	Relaxation Delay	5.0000
13	Pulse Width	4.7500
14	Acquisition Time	2.1863
15	Acquisition Date	2017_03_20 T10:42:10
16	Modification Date	2017_03_20 T10:43:17
17	Spectrometer Frequency	399.78
18	Spectral Width	5995.2
19	Lowest Frequency	_998.7
20	Nucleus	1H
21	Acquired Size	16384
22	Spectral Size	52430

S2. ^{13}C-NMR spectrum (100 MHz) of Dothiorelone A (1) in CD_3OD.

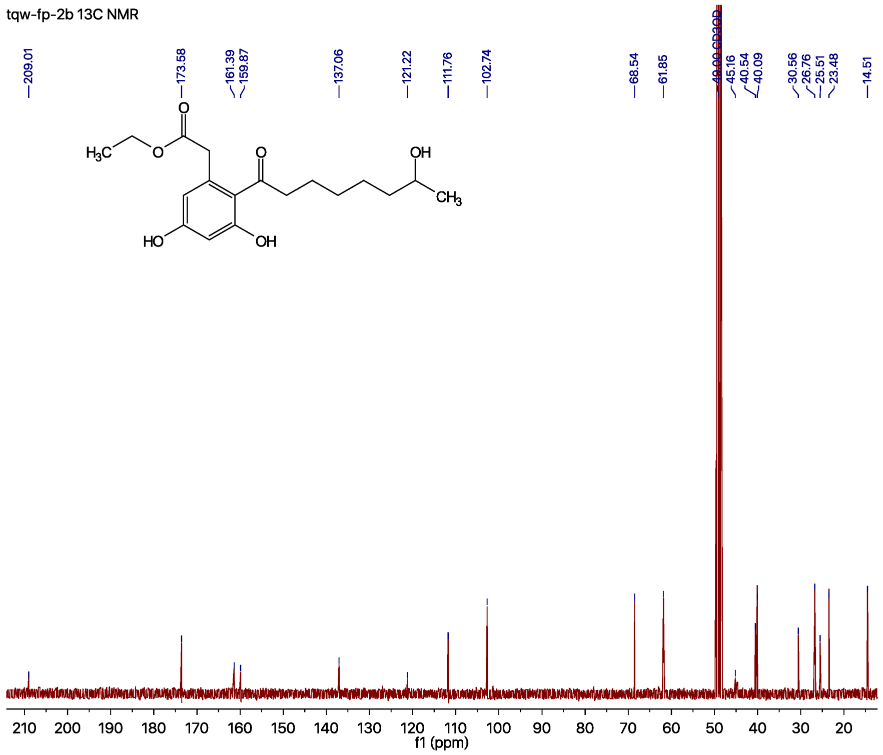

	Parameter	Value
1	Title	FP_2b
2	Origin	JEOL
3	Spectrometer	ECA
4	Author	delta
5	Solvent	METHANOL_D4
6	Temperature	23.7
7	Pulse Sequence	carbon.jxp
8	Experiment	1D
9	Probe	2756
10	Number of Scans	10240
11	Receiver Gain	50.0
12	Relaxation Delay	2.0000
13	Pulse Width	3.3333
14	Acquisition Time	1.0381
15	Acquisition Date	2017_03_20T 10:48:33
16	Modification Date	2017_03_20T 19:29:02
17	Spectrometer Frequency	100.53
18	Spectral Width	25252.5
19	Lowest Frequency	_2573.7
20	Nucleus	13C
21	Acquired Size	32768
22	Spectral Size	52430

S3. ^{1}H-NMR spectrum (400 MHz) of Dothiorelone A (1) in CD_3OD.

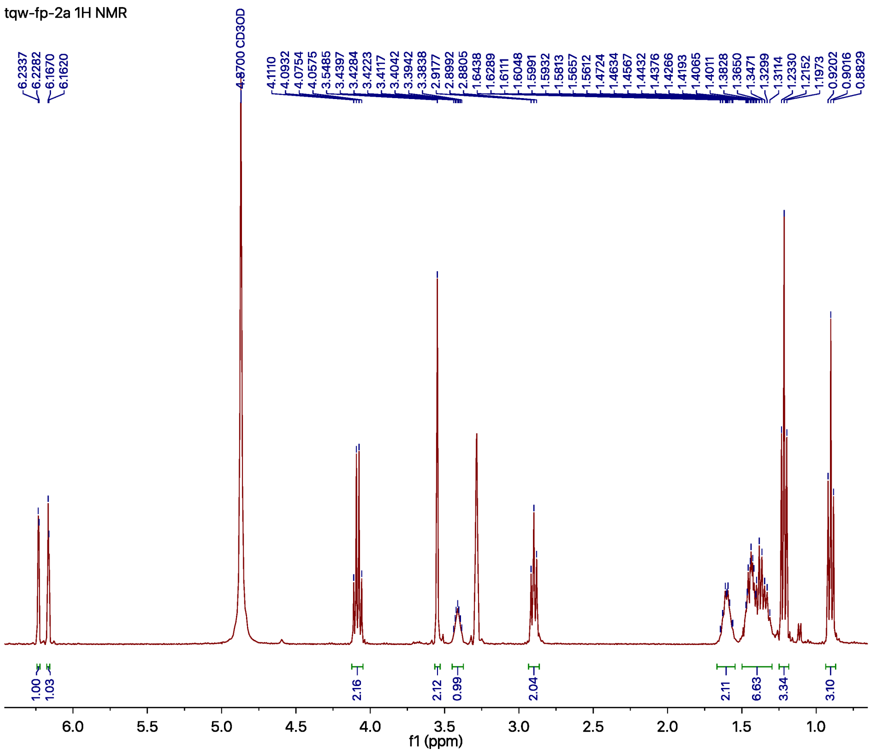

	Parameter	Value
1	Title	fp_2A
2	Origin	JEOL
3	Spectrometer	ECA
4	Author	delta
5	Solvent	METHANOL_D4
6	Temperature	20.9
7	Pulse Sequence	proton.jxp
8	Experiment	1D
9	Probe	2756
10	Number of Scans	8
11	Receiver Gain	56.0
12	Relaxation Delay	5.0000
13	Pulse Width	4.7500
14	Acquisition Time	2.1863
15	Acquisition Date	2017_03_11T 20:22:21
16	Modification Date	2017_03_11T 20:23:30
17	Spectrometer Frequency	399.78
18	Spectral Width	5995.2
19	Lowest Frequency	_998.7
20	Nucleus	1H
21	Acquired Size	16384
22	Spectral Size	52430

S4. ^{13}C-NMR spectrum (100 MHz) of Dothiorelone A (1) in CD_3OD.

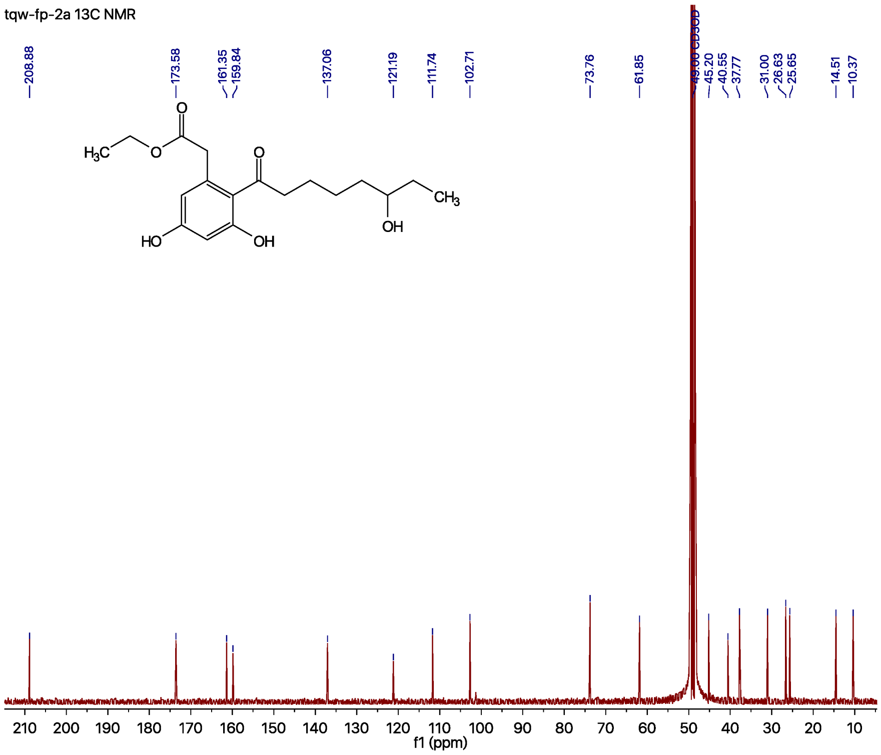

	Parameter	Value
1	Title	fp_2A
2	Origin	JEOL
3	Spectrometer	ECA
4	Author	delta
5	Solvent	METHANOL_D4
6	Temperature	21.1
7	Pulse Sequence	carbon.jxp
8	Experiment	1D
9	Probe	2756
10	Number of Scans	1024
11	Receiver Gain	50.0
12	Relaxation Delay	2.0000
13	Pulse Width	3.3333
14	Acquisition Time	1.0381
15	Acquisition Date	2017_03_11T 20:24:33
16	Modification Date	2017_03_11T 21:16:51
17	Spectrometer Frequency	100.53
18	Spectral Width	25252.5
19	Lowest Frequency	_2573.7
20	Nucleus	13C
21	Acquired Size	32768
22	Spectral Size	52430

S5. ^{1}H-NMR spectrum (400 MHz) of Dothiorelone C (3) in CD_3OD.

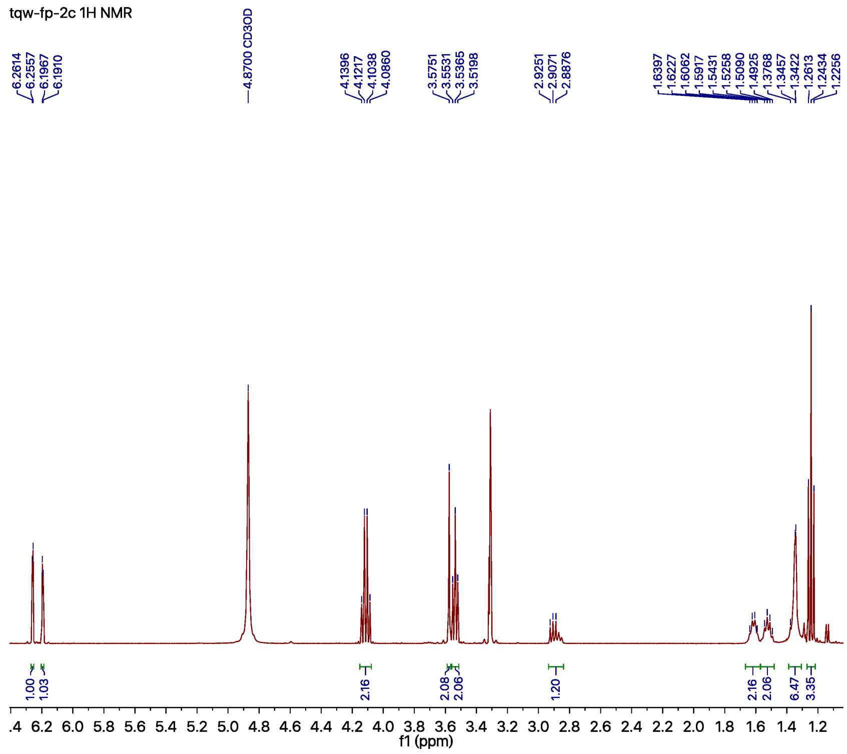

	Parameter	Value
1	Title	FP_2C
2	Origin	JEOL
3	Spectrometer	ECA
4	Author	delta
5	Solvent	METHANOL_D4
6	Temperature	23.3
7	Pulse Sequence	proton.jxp
8	Experiment	1D
9	Probe	2756
10	Number of Scans	8
11	Receiver Gain	56.0
12	Relaxation Delay	5.0000
13	Pulse Width	4.7500
14	Acquisition Time	2.1863
15	Acquisition Date	2017_03_20T20:15:33
16	Modification Date	2017_03_20T20:16:40
17	Spectrometer Frequency	399.78
18	Spectral Width	5995.2
19	Lowest Frequency	_998.7
20	Nucleus	1H
21	Acquired Size	16384
22	Spectral Size	52430

S6. ^{13}C-NMR spectrum (100 MHz) of Dothiorelone C (3) in CD_3OD.

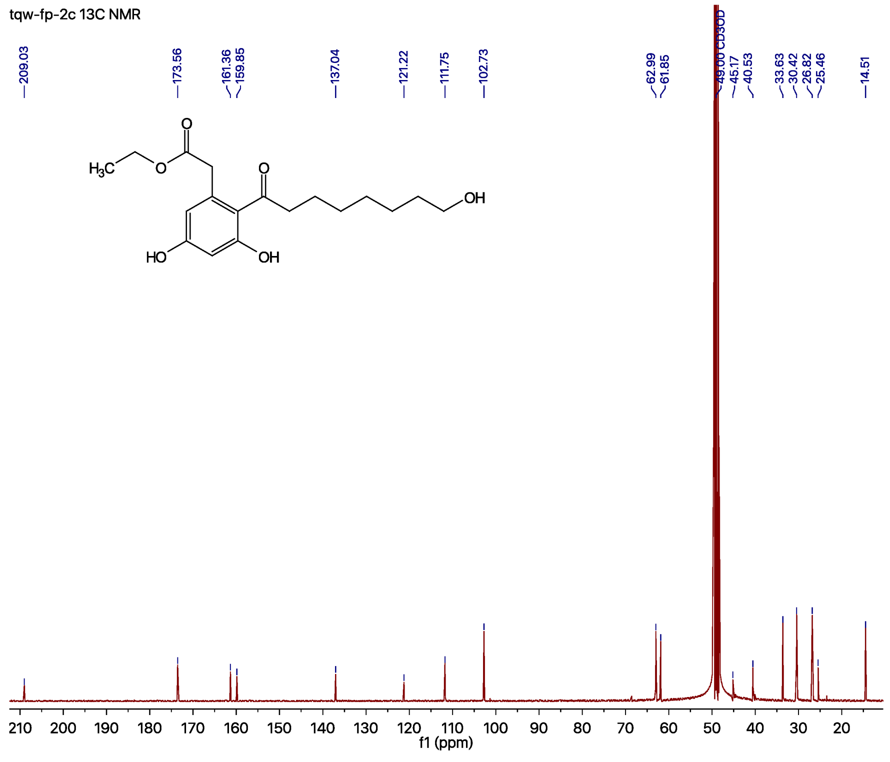

	Parameter	Value
1	Title	FP_2C
2	Origin	JEOL
3	Spectrometer	ECA
4	Author	delta
5	Solvent	METHANOL_D4
6	Temperature	23.1
7	Pulse Sequence	carbon.jxp
8	Experiment	1D
9	Probe	2756
10	Number of Scans	15024
11	Receiver Gain	50.0
12	Relaxation Delay	2.0000
13	Pulse Width	3.3333
14	Acquisition Time	1.0381
15	Acquisition Date	2017_03_20T20:17:54
16	Modification Date	2017_03_21T09:01:26
17	Spectrometer Frequency	100.53
18	Spectral Width	25252.5
19	Lowest Frequency	_2573.7
20	Nucleus	13C
21	Acquired Size	32768
22	Spectral Size	52430

S7. ^{1}H-NMR spectrum (500 MHz) of Dothiorelone H (4) in CD_3OD.

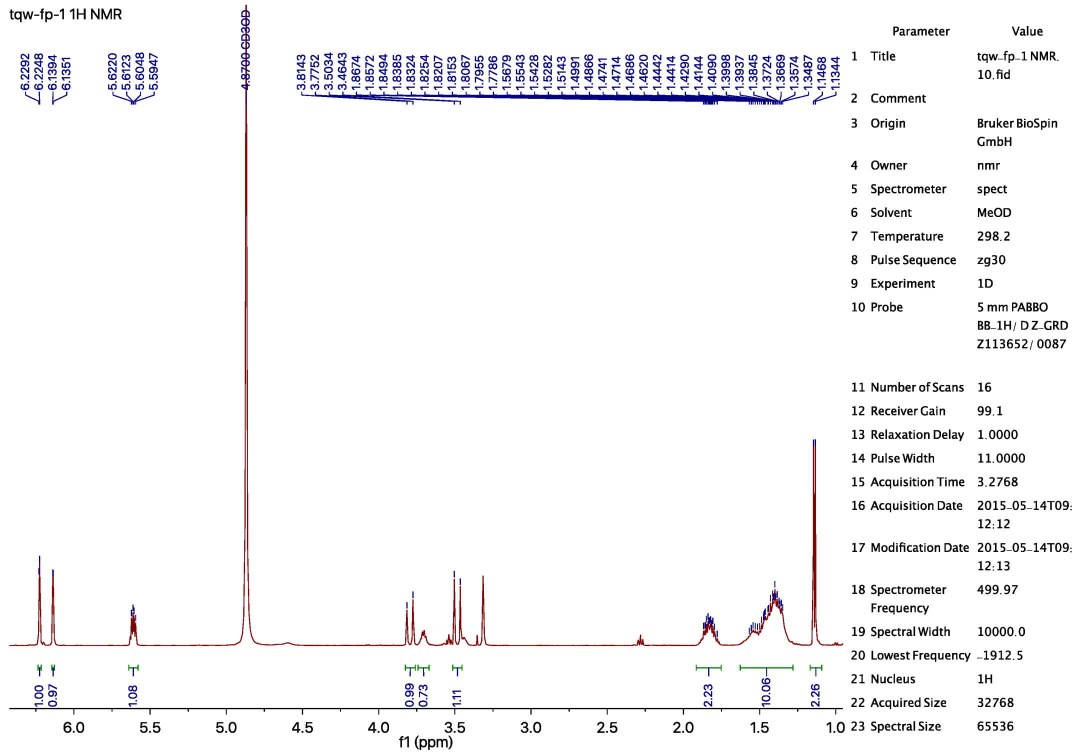

S8. ^{13}C-NMR spectrum (125 MHz) of Dothiorelone H (4) in CD_3OD.

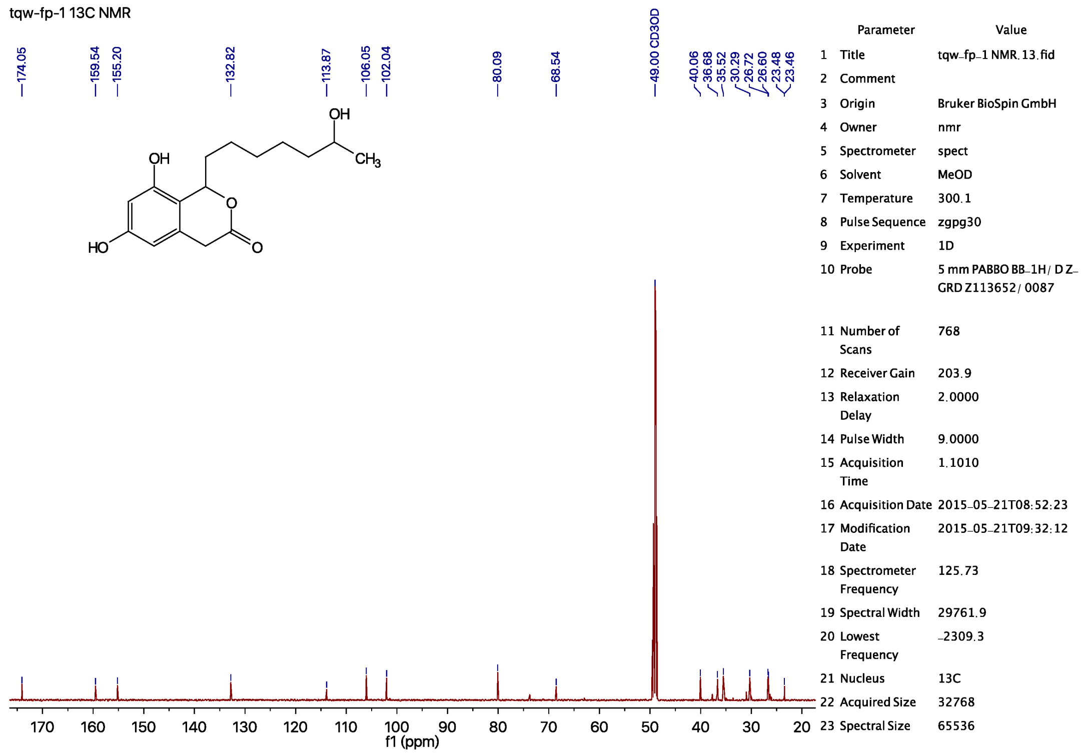

S9. ^{1}H-NMR spectrum (500 MHz) of Cytosporone C (5) in CD_3OD.

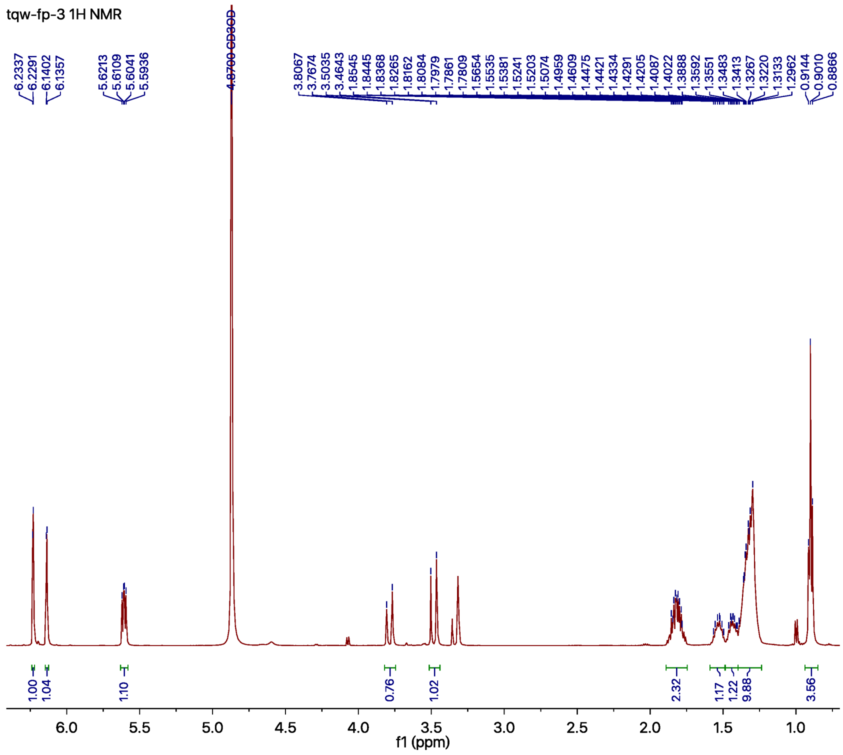

	Parameter	Value
1	Title	tqw-fp-3 NMR.10.fid
2	Comment	
3	Origin	Bruker BioSpin GmbH
4	Owner	nmr
5	Spectrometer	spect
6	Solvent	MeOD
7	Temperature	298.1
8	Pulse Sequence	zg30
9	Experiment	1D
10	Probe	5 mm PABBO BB-1H/ D Z-GRD Z113652/ 0087
11	Number of Scans	16
12	Receiver Gain	99.1
13	Relaxation Delay	1.0000
14	Pulse Width	11.0000
15	Acquisition Time	3.2768
16	Acquisition Date	2015-05-14T09:21:01
17	Modification Date	2015-05-14T09:21:02
18	Spectrometer Frequency	499.97
19	Spectral Width	10000.0
20	Lowest Frequency	-1912.5
21	Nucleus	1H
22	Acquired Size	32768
23	Spectral Size	65536

S10. ^{13}C-NMR spectrum (125 MHz) of Cytosporone C (5) in CD_3OD.

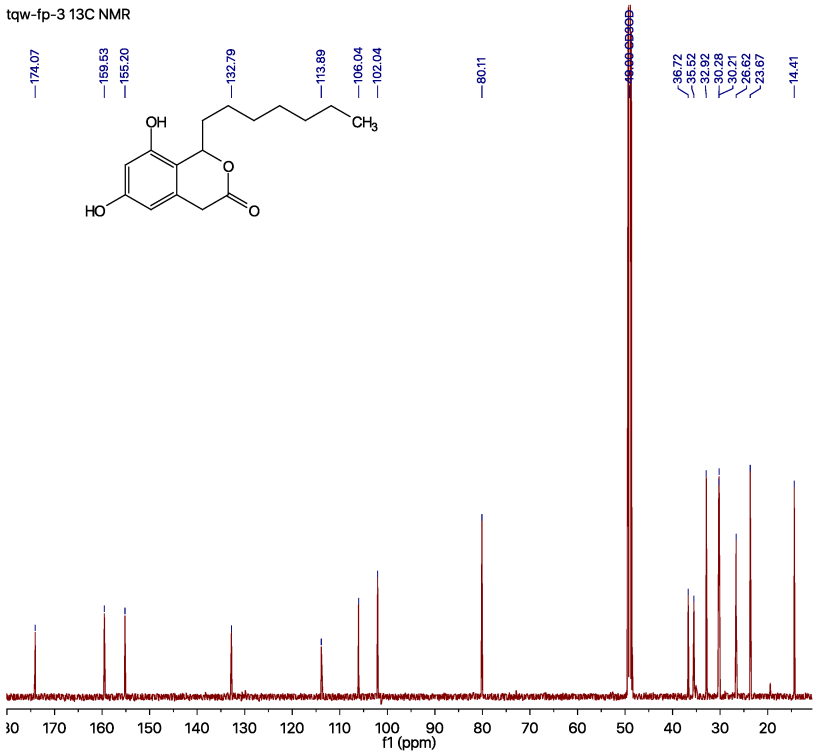

	Parameter	Value
1	Title	tqw-fp-3 NMR.13.fid
2	Comment	
3	Origin	Bruker BioSpin GmbH
4	Owner	nmr
5	Spectrometer	spect
6	Solvent	MeOD
7	Temperature	299.3
8	Pulse Sequence	zgpg30
9	Experiment	1D
10	Probe	5 mm PABBO BB-1H/ D Z-GRD Z113652/ 0087
11	Number of Scans	512
12	Receiver Gain	203.9
13	Relaxation Delay	2.0000
14	Pulse Width	9.0000
15	Acquisition Time	1.1010
16	Acquisition Date	2015-05-21T10:20:31
17	Modification Date	2015-05-21T10:47:19
18	Spectrometer Frequency	125.73
19	Spectral Width	29761.9
20	Lowest Frequency	-2309.3
21	Nucleus	13C
22	Acquired Size	32768
23	Spectral Size	65536

S11. ^{1}H-NMR spectrum (500 MHz) of Cytosporone U (6) in CD_3OD.

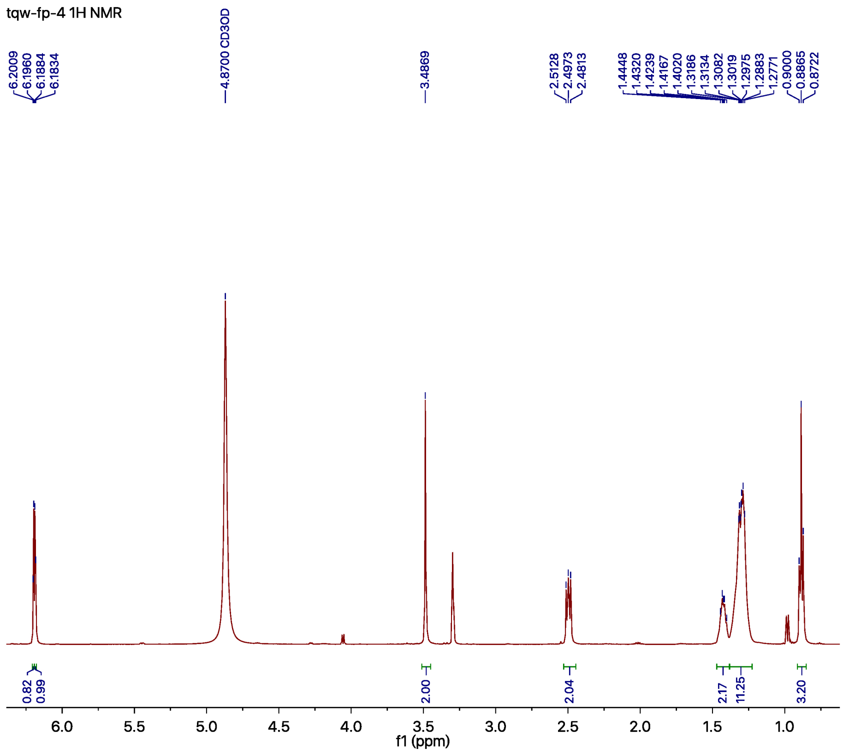

	Parameter	Value
1	Title	tqw-fp-4 NMR. 10.fid
2	Comment	
3	Origin	Bruker BioSpin GmbH
4	Owner	nmr
5	Spectrometer	spect
6	Solvent	MeOD
7	Temperature	298.1
8	Pulse Sequence	zg30
9	Experiment	1D
10	Probe	5 mm PABBO BB-1H/ D Z-GRD Z113652/ 0087
11	Number of Scans	16
12	Receiver Gain	99.1
13	Relaxation Delay	1.0000
14	Pulse Width	11.0000
15	Acquisition Time	3.2768
16	Acquisition Date	2015-05-14T09:25:40
17	Modification Date	2015-05-14T09:25:41
18	Spectrometer Frequency	499.97
19	Spectral Width	10000.0
20	Lowest Frequency	-1912.5
21	Nucleus	1H
22	Acquired Size	32768
23	Spectral Size	65536

S12. ^{13}C-NMR spectrum (125 MHz) of Cytosporone U (6) in CD_3OD.

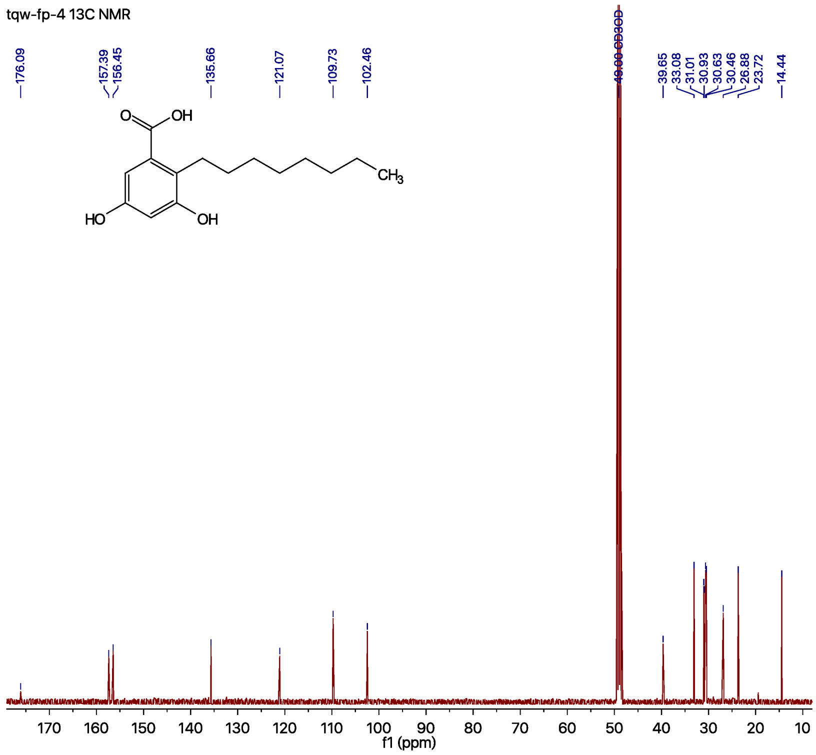

	Parameter	Value
1	Title	tqw-fp-4 NMR.13.fid
2	Comment	
3	Origin	Bruker BioSpin GmbH
4	Owner	nmr
5	Spectrometer	spect
6	Solvent	MeOD
7	Temperature	299.7
8	Pulse Sequence	zgpg30
9	Experiment	1D
10	Probe	5 mm PABBO BB-1H/ D Z-GRD Z113652/ 0087
11	Number of Scans	512
12	Receiver Gain	203.9
13	Relaxation Delay	2.0000
14	Pulse Width	9.0000
15	Acquisition Time	1.1010
16	Acquisition Date	2015-05-21T10:51:31
17	Modification Date	2015-05-21T11:17:44
18	Spectrometer Frequency	125.73
19	Spectral Width	29761.9
20	Lowest Frequency	-2309.3
21	Nucleus	13C
22	Acquired Size	32768
23	Spectral Size	65536

S13. ^{1}H-NMR spectrum (500 MHz) of the purified polysaccharide in D_2O.

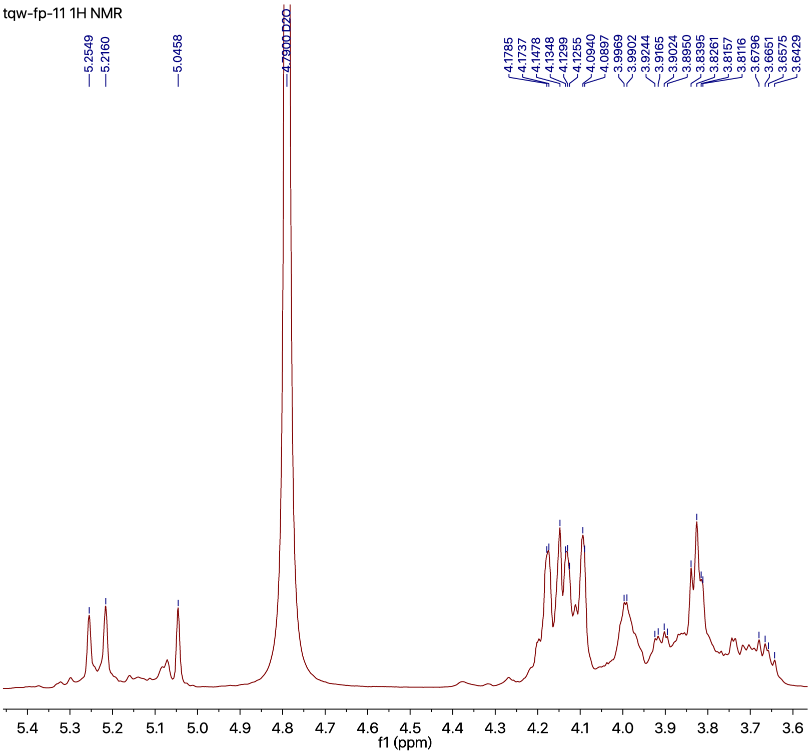

	Parameter	Value
1	Title	tqw-fp-15.10.fid
2	Comment	1H
3	Origin	Bruker BioSpin GmbH
4	Spectrometer	spect
5	Solvent	D2O
6	Temperature	299.9
7	Pulse Sequence	zg30
8	Experiment	1D
9	Probe	5 mm PABBO BB-1H/ D Z-GRD Z113652/ 0087
10	Number of Scans	16
11	Receiver Gain	31.1
12	Relaxation Delay	1.0000
13	Pulse Width	12.6000
14	Acquisition Time	3.2768
15	Acquisition Date	2017-10-13T08:29:36
16	Modification Date	2017-10-13T08:29:38
17	Spectrometer Frequency	500.13
18	Spectral Width	10000.0
19	Lowest Frequency	-1911.5
20	Nucleus	1H
21	Acquired Size	32768
22	Spectral Size	65536

S14. ^{13}C-NMR spectrum (125 MHz) of the purified polysaccharide in D_2O.

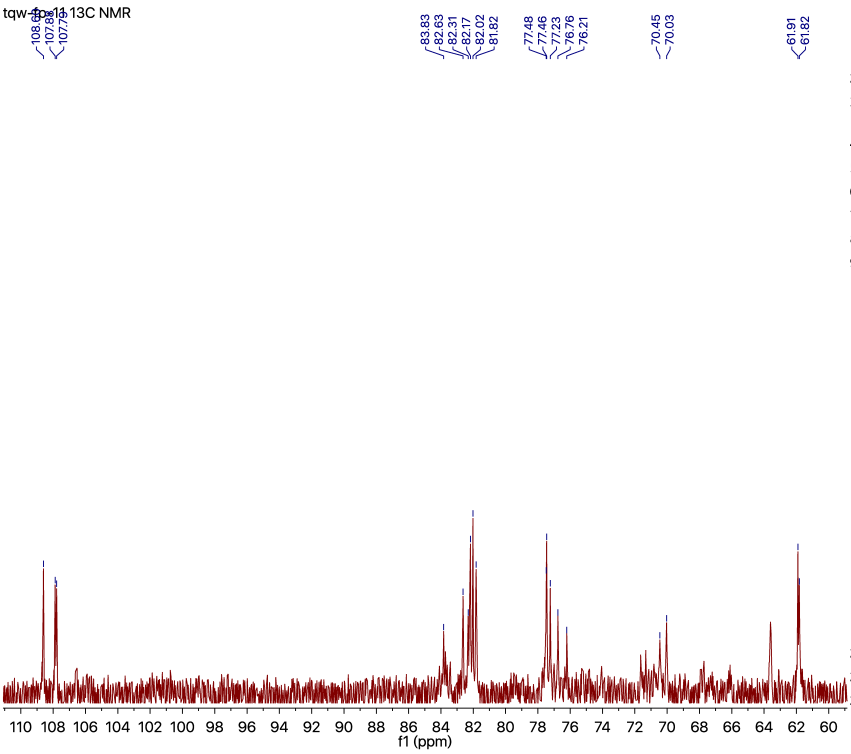

	Parameter	Value
1	Title	tqw-fp-15.13.fid
2	Comment	13C
3	Origin	Bruker BioSpin GmbH
4	Spectrometer	spect
5	Solvent	D2O
6	Temperature	300.4
7	Pulse Sequence	zgpg30
8	Experiment	1D
9	Probe	5 mm PABBO BB-1H/ D Z-GRD Z113652/ 0087
10	Number of Scans	1024
11	Receiver Gain	203.9
12	Relaxation Delay	2.0000
13	Pulse Width	9.5000
14	Acquisition Time	1.1010
15	Acquisition Date	2017-10-13T08:43:11
16	Modification Date	2017-10-13T09:36:52
17	Spectrometer Frequency	125.77
18	Spectral Width	29761.9
19	Lowest Frequency	-2305.8
20	Nucleus	13C
21	Acquired Size	32768
22	Spectral Size	65536

S15. HSQC spectrum of the purified polysaccharide.

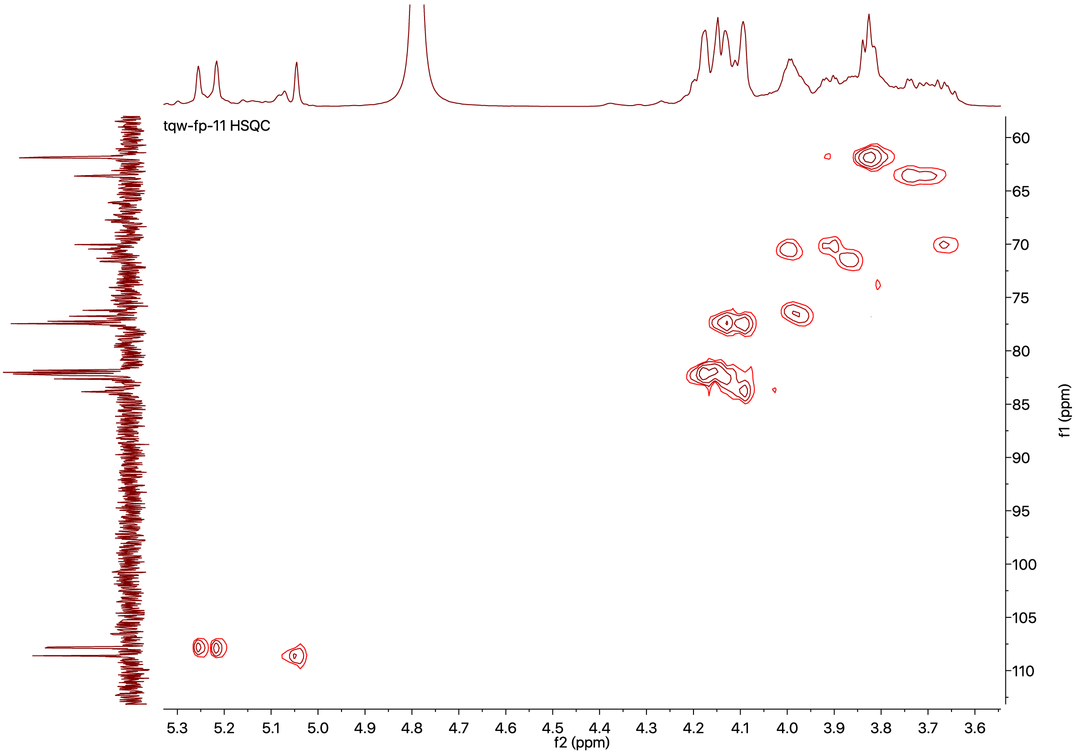

S16. TOSCY spectrum of the purified polysaccharide.

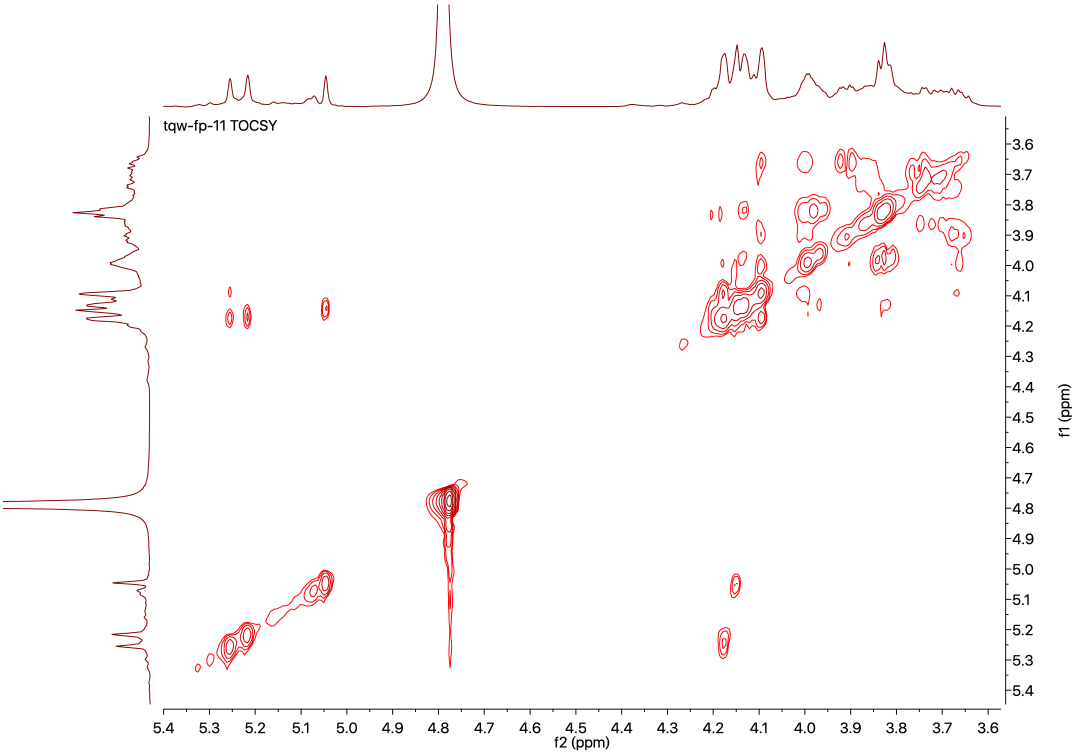

S17. NOESY spectrum of the purified polysaccharide.

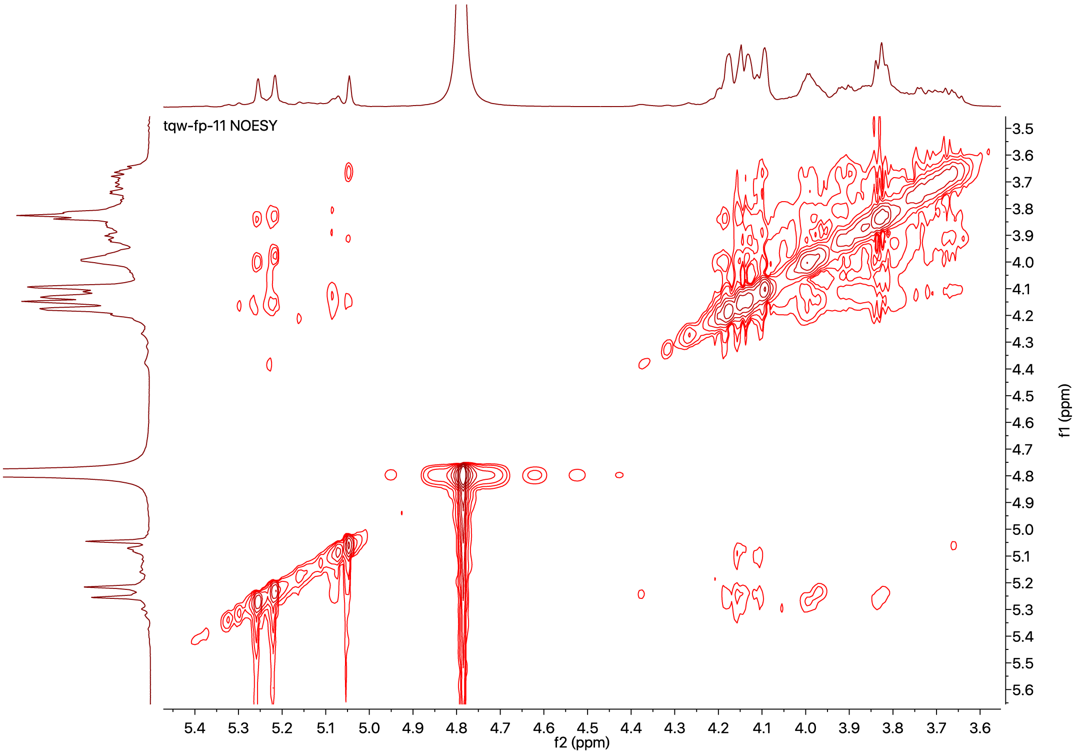

Supplement: Supplementary file 1 [file molecules-22-02073-s001.pdf]
